# The temperature of internet: Internet use and depression of the elderly in China

**DOI:** 10.3389/fpubh.2022.1076007

**Published:** 2022-12-21

**Authors:** Hongwang Guo, Shuyi Feng, Ziming Liu

**Affiliations:** ^1^School of Public Administration and Policy, Renmin University of China, Beijing, China; ^2^China Resources, Environment and Development Academy, Nanjing Agricultural University, Nanjing, China; ^3^School of Social and Public Administration, East China University of Science and Technology, Shanghai, China

**Keywords:** internet use, mental health, propensity score matching, endogenous switching regression, depression

## Abstract

**Introduction:**

Depression has become one of the most prevalent mental illnesses affecting the elderly in aging countries, i. e., in countries of the world whose population is slowly aging. It has become an important topic for scientists and policymakers to analyze how best to improve the elderly's mental health and save them from depression. The aim of this paper was to investigate whether and to what extent internet use may affect depression in the elderly. The heterogeneous effects of internet use on the elderly's depression across age, gender, and occupation were also investigated.

**Methods:**

The data used in the present study were gathered from the China Health and Retirement Longitudinal Study that was conducted in 2018. The propensity score matching technique and the endogenous switch regression model were employed in this study to address potential endogeneity caused by both observed and unobserved factors.

**Results:**

The results of the present study show that the elderly who are relatively young, male, well educated, live in an urban area, or have a small family are more likely to use the internet. The elderly who have healthy eyes or good eyesight, those who are not employed in the agricultural sector, or those who are retired, and those who are not eligible to receive any subsistence allowance or drink wine have a higher probability of using the internet. We also find that internet use significantly reduces the elderly's depression status by 3.370 points, which is roughly equivalent to a reduction of 37.19%. Heterogeneity analysis on internet use reveals that the health effect is particularly effective for agricultural workers, female, or the older elderly.

**Conclusion:**

The results of the present study highlight the significant welfare effects brought about by the development of internet infrastructure. To improve the mental health of the elderly, the government should encourage them to adopt the internet. In particular, the needs of the elderly who are older, female, or have agricultural work should be paid more attention to motivate them to use the internet more to alleviate depression.

## Introduction

Various health problems faced by several elderly people have attracted a lot of attention from people all around the globe, including scientists and policymakers. As regards the elderly, deterioration in physical function, disability, chronic diseases, cognitive impairment, and other health problems seriously reduce the quality of their life and increase medical expenditure of their families (1). As the population of aged people continues to grow so do their health problems, putting tremendous pressure on social insurance programs to meet the rising costs of the elderly's health problems, which may further undermine a country's economic development (2). China, as one of the most populated nations of the world, has the largest elderly population and probably the highest old-age dependency ratio in the world (3). The Chinese seventh census survey shows that the Chinese population over the age of 60 years exceeded 260 million in 2020, which accounted for ~18.7% of the total population.

Although depression has become one of the most prevalent mental illnesses affecting the elderly, it has in fact received much less attention than other health issues (4). It is reported that ~17–27% of the Chinese elderly suffer from depression, which is a much higher value than the values observed in many other countries of the world (5). Depression reduces the elderly's happiness and satisfaction in life, and even causes a number of other health problems such as chronic diseases and provoking suicidal thoughts, thereby bringing a heavy burden to the elderly's families and the society at large (6). Thus, it has become an important topic for both scientists and policymakers alike to study in depth how to reduce the likelihood of depression among the elderly and save them from depression.

While we can attribute various causes for depression among the elderly groups, the lack of social participation serves as an important cause for triggering depression (7–9). For example, empirical evidence shows that participation by the elderly in leisure activities helps to improve their mental health to a large extent (10). In particular, engaging them in leisure social participation such as playing cards or chess has a remarkable effect on their mental wellbeing. However, social participation is not confined to specific forms. The form of people's social participation may affect mental health differently. For the elderly, continuous social participation often has a larger inhibitory effect on depression than occasional participation (7, 11). While voluntary participation in community activities may have positive effect on the elderly's mental health, forced participation may bring about negative effects (12).

In particular, the use of internet is often recognized as a vital form of social participation (13). With the progress in internet technology and the intensification of the aging process, the elderly groups have become an important force to reckon with among internet users (14). Indeed, information technology assists the elderly who struggle from physical inconvenience to shop, socialize, and form close relationships with the society (15). As a simple, convenient, and low-cost means of social participation, internet use reduces the incidence of depression (16, 17).

In this paper, we propose that there are at least two mechanisms through which internet use may drastically alter the elderly's depression status. First, internet use may allow the elderly to maintain the hitherto cordial relationship with relatives and friends, or even establish an alternative relationship network by striking a rapport with new friends (18). Indeed, usually the elderly's access to social participation is often restricted due to their nagging physical health issue (19). But internet comes to their succor by dispensing with such restrictions that arise from space, cost, and other conditions that are very much required for any social participation, and thereby allows the elderly to communicate hassle-free with the outside world. In fact, such a means of communication plays an important role in delaying anxiety, improving their sense of belonging, and reducing their loneliness, and it is equally effective in reducing depressive symptoms (20). In China, where the so-called “empty nest elderly” are popular, the use of internet can effectively reduce their loneliness and life pressure (21).

Second, internet use may maintain or improve the elderly's cognitive ability, which is an important predictor of depression (22). The common logic behind use of the internet is that its use allows the elderly to retrieve the hitherto gained memory on disease prevention and health-related knowledge, which indirectly improves their health status (23). In addition, active internet use requires the elderly to conduct a number of mental activities, such as to search, to think, or to calculate, which train the elderly's brain frequently and eventually improve their cognitive ability. Evidence from an experiment shows that training the elderly to use tablet devices increases their episodic memory and data processing ability (16). Another study finds that internet use lowers the prevalence of dementia in the elderly (24).

However, if internet use were too frequent, bordering the case of internet addiction, the internet use may then worsen the depression status of the elderly. When we generally speak of internet addiction, we usually associate it with young people only. For example, existing literature shows that internet addiction is often positively associated with the incidence of depression among the youngsters (25, 26). Yet, for the elderly mentioned in our paper we do not think internet addiction is applicable. Indeed, in China, internet addiction mainly occurs in the younger groups, e.g., teenagers and students (27). Nevertheless, in this paper, we don't think that internet addiction has a significant impact on the elderly. Overall, we expect internet use to lower the prevalence of depression in the elderly in China.

The objective of this paper was to investigate whether and to what extent internet use may affect the mental health of the elderly. The data used in the study were drawn from the China Health and Retirement Longitudinal Study (CHARLS) conducted in 2018. We employ the propensity score matching (PSM) technique to address the concern of selection bias caused by the observed factors. The endogenous switching regression (ESR) model is employed to further address the potential selection bias due to factors which are unobservable. We also examine the heterogeneous effects of internet use on the elderly across age, gender, and occupation.

## Methods

### Data

This paper uses the data from the China Health and Retirement Longitudinal Survey (CHARLS) 2018 for analysis. The CHARLS project is led by the Peking University, which aims to collect a large and representative sample of the population aged 45 years and above in China. Specifically, the CHARLS 2018 covers ~150 counties, 450 communities or villages in China, and consists of ~23,000 respondents in 28 provinces. The sample was selected following the probability proportional to the size sampling strategy. Due to their high-quality nature, these data have been widely used in scientific research and have generated many publications.

In the questionnaire, 10 questions from the CES-D (Center for Epidemiological Studies-Depression) are designed and drawn up for the depression test (Supplementary Table A1). For example, the respondent was asked if in the past week he/she was bothered, depressed, fearful, lonely, or always had trouble in mind, felt hard to do anything, could not sleep well or get going. The answer options for each question were “little or no,” “not much,” “sometimes or half the time,” and “most of the time,” which were assigned with values of 0, 1, 2, and 3, respectively. Out of the 10, there were two opposite questions, i.e., “I am full of hope for the future” and “I am very happy,” with the same answer options. We used the reverse scoring method for the answers to the two questions. The scores of the 10 questions were then summed to generate the CES-D score as the outcome variable. The respondent with a CES-D score ≥10 is often defined as having symptoms of depression (28). Our results show that the overall incidence of depression among the Chinese elderly was ~33.84% in 2018.

Besides the module of health status, the questionnaire also collected information about individual and family characteristics, e.g., age, gender, education, marriage status, family size, living habits, and daily activities. In particular, a question on learning about the respondent's behavior of internet use is designed. In that question, the respondent was asked if he/she had used the internet in the past month, with two answer options of “yes” or “no.” We used the information from this question to generate our explanatory variable. During the data cleaning process, we excluded those observations of persons whose age was below 45 years. We also removed those observations that had missing values in our selected variables. The final sample for analysis in this paper has 17,365 observations.

### Empirical strategies

A. Propensity score matching

The major challenge in identifying the effect of internet use on the elderly's depression status pertains to the fact that the elderly's behavior of internet use could be endogenous, i.e., internet use and depression might be affected by many common factors. This endogenous behavior on the part of the elderly may result in the concern of selection bias, if internet users are compared to non-users directly (29). To address such concern of selection bias, we employed the propensity score matching (PSM) technique. The PSM technique eliminates the impact of covariates by resembling the randomized assignment to treatment, to create conditions of a random experiment (30). The PSM has been widely employed in empirical work (31–33). Specifically, the following probit model is estimated in the first stage:


(1) $$INT_{i}^{*}=X_{i}\alpha +\varepsilon_{i}\text{ with }INT_{i}=\left\{ \begin{array}{l} 1\;if\;INT_{i}^{*}>0\\ 0\;otherwise \end{array}\right.$$

where $INT_{i}^{*}$ is a latent variable that indicates the utility of respondent *i*'s choice of internet use. If the utility exceeds 0, we observe that the respondent chooses to use the internet (*INT*_*i*_ = 1); otherwise, the respondent does not use the internet (*INT*_*i*_ = 0). *X*_*i*_ is a set of exogenous variables, which affects the respondent's choice of internet use. Estimation from Equation (1) reports the determinants of the elderly's choice of internet use and allows us to predict the propensity score of each observation to use the internet.

The predicted propensity scores are then used to find one or more matching partners for internet users from non-users. We use the popular matching algorithms, i.e., nearest neighbor, radius, and kernel matching, to find matching partners (34). Taking advantage of the large sample, we select one, five, and ten matching partners in the nearest neighbor matching for robustness tests. A matching caliper of 0.001 is set to reduce potential matching bias. After matching, we compute the average treatment effect on the treated (ATT) of internet use on depression status following Equation (2):


(2) $$\begin{array}{l} ATT=E\left(Y_{1}|INT_{i}=1\right)-E\left(Y_{0}|INT_{i}=1\right) \end{array}$$

where *Y*_1_ and *Y*_0_ are the elderly's depression statuses of the matched internet users and non-users, respectively.

The validity of PSM depends on three assumptions. First, before matching there must be a sufficient overlap of propensity scores between internet users and non-users. Second, after matching, the covariates must statistically have no difference between internet users and non-users. Third, in the selection function there is no omitted variable that is correlated with internet use and the elderly's depression status (35). In the Results section, we present supportive evidence for the satisfaction of these assumptions.

B. Endogenous switching regression

A major problem of the PSM is that it can only mitigate selection bias due to observables but not due to unobservables. We therefore employ the endogenous switching regression (ESR) model, which accounts for selection bias from both observables and unobservables, to complement the PSM. The ESR model has also been widely employed in empirical work (36–39). The typical ESR model has three equations. The first equation, as shown in Equation (1), determines two regimes that a respondent may fall into, namely, using the internet or not. The rest of the two equations explain the outcome variable, i.e., the depression status, under different regimes:


(3a) $$\begin{array}{l} \text{Regime }1:\;Y_{1i}=Z_{i}\beta_{1}+\eta_{1i}\text{ if }INT_{i}=1 \end{array}$$

(3b) $$\begin{array}{l} \text{Regime }2:\;Y_{0i}=Z_{i}\beta_{0}+\eta_{0i}\text{ if }INT_{i}=0 \end{array}$$

where *Y*_1*i*_ and *Y*_0*i*_ are the measures of a respondent's depression status, which are observed only under regimes one and two, respectively. *Z*_*i*_ contains all the variables in *X*_*i*_ and at least one instrumental variable. The error terms ε_*i*_, η_1*i*_, and η_0*i*_ presumptively follow a joint normal distribution. In the estimation process, two inverse Mills ratios λ_1*i*_ and λ_0*i*_ are predicted using Equation (1) for internet users and non-users, respectively. The outcome equations are then updated by including the inverse Mills ratios:


(4a) $$\begin{array}{l} \text{Regime }1:\;Y_{1i}=Z_{i}\beta_{1}+{\text{λ}}_{1i}\delta_{1}+\eta_{1i}\text{ if }INT_{i}=1 \end{array}$$

(4b) $$\begin{array}{l} \text{Regime }2:\;Y_{0i}=Z_{i}\beta_{0}+{\text{λ}}_{0i}\delta_{0}+\eta_{0i}\text{ if }INT_{i}=0 \end{array}$$

where δ_1_ and δ_0_ are the parameters to be estimated for the inverse Mills ratios. To estimate the selection and outcome equations simultaneously, a full information maximum-likelihood method should be employed (40). The estimated parameters in the outcome equations are then used to predict the expected depression status.


(5a) $$\begin{array}{l} E\left(Y_{1i}|INT_{i}=1\right)=Z_{i}\beta_{1}+{\text{λ}}_{1i}\delta_{1} \end{array}$$

(5b) $$\begin{array}{l} E\left(Y_{0i}|INT_{i}=1\right)=Z_{i}\beta_{0}+{\text{λ}}_{0i}\delta_{0} \end{array}$$

where *E*(*Y*_1*i*_|*INT*_*i*_ = 1) is the expected depression status of the elderly who use internet and *E*(*Y*_0*i*_|*INT*_*i*_ = 1) is the expected depression status of the elderly in the counterfactual scenario. The average treatment effect on the treated is then computed following Equation (6):


(6) $$\begin{array}{l} \text{ATT}=E\left(Y_{1i}|INT_{i}=1\right)-E\left(Y_{0i}|INT_{i}=1\right) \end{array}$$

Heterogeneous effects of internet use on depression status are estimated by restricting Equation (6) to sub-group samples.

C. Variable definitions

Table 1 presents the variable definitions. The outcome variable is the CES-D score, which is a reliable and widely used measure of the depression status in clinical practices and epidemiological studies (41, 42). The explanatory variable is internet use, defined as 1 if the respondent used the internet in the last month, and 0 otherwise.

**Table 1:** Variable definitions.

**Variables**	**Definitions**
Depression	The score was calculated according to the CES-D scale
Internet use	1 = use the Internet; 0 = not use the Internet
Age	Age of respondents
Male	1 = male; 0 = female
Urban	1 = have a Urban Hukou; 0 = otherwise
Primary	1 = if the highest education is primary school or below; 0 = otherwise
Junior	1 = if the highest education is junior high school; 0 = otherwise
Senior	1 = if the highest education is senior high school or above; 0 = otherwise
Family size	The number of family members
Married	1 = married; 0 = not married
Sleep time	The time when you sleep every night (hours)
Vision	1 = good vision; 0 = otherwise
Agricultural work	1 = engaged in agricultural work; 0 = otherwise
Retirement	1 = withdrawal from the labor market; 0 = otherwise
Subsistence allowance	1 = subsidized; 0 = no subsidy
Drinking	1 = ever smoked before; 0 = otherwise
Smoking	1 = ever drank alcohol last year; 0 = otherwise

Authors' own design.

The variables included in the selection function are important. Some scholars propose that the variables should be theoretically important for outcomes (43). Whereas some other scholars argue that they should be simultaneously associated with selection and outcomes (34). In practice, empirical studies using PSM may choose variables that are important for selection (44), outcomes (45), or both (30, 46). In this paper, we follow the literature and select a set of variables, e.g., age, gender, education, martial status, family size, and living habits, which are associated with internet use and mental health to better predict the choice of internet use and mental health (47, 48).

The selected instrumental variable is the ratio of internet use by other elderly in the same communities. We propose that the ratio of internet use by other elderly in the same communities should be correlated with a respondent's behavior of internet use due to the peer effect (49). Besides, internet use by other people is simply their personal behavior, which is unlikely to have an impact on a specific elderly's depression scores. Such choice of instrumental variable has been proved valid in many empirical studies (30, 50, 51).

## Results

### Descriptive analysis

**Figure 1:**
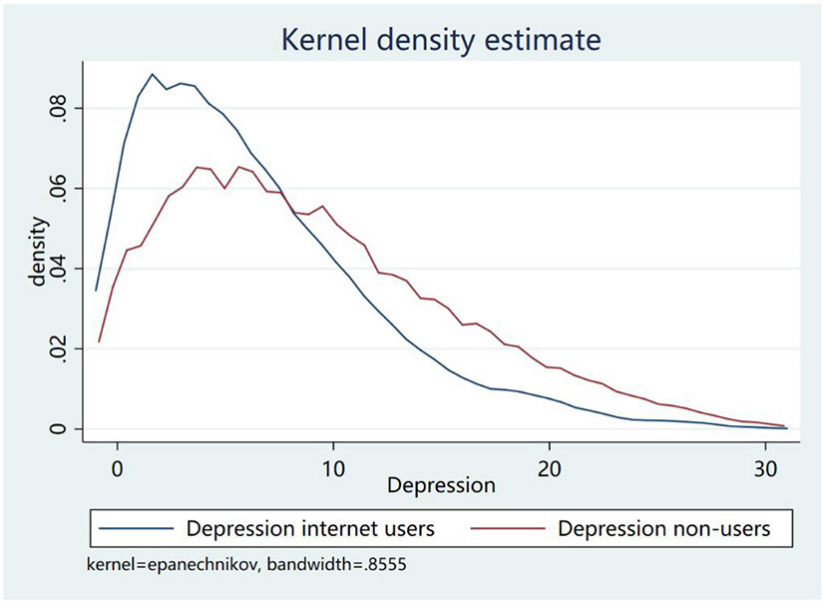
Depression scores between internet users and non-users.

Figure 1 shows the differences in depression scores between internet users and non-users. In general, the depression scores of the elderly who use the internet are smaller than those of the non-users. Table 2 further reports differences in the average depression scores between the elderly who are internet users and non-users. We find that the average CES-D score for the full sample is 8.709, while for the sub-samples of the internet users and non-users, the average CES-D scores are 6.374 and 9.061, respectively. That is, compared with the elderly who do not use the internet, those who use the internet score lower in the depression measure. The difference in depression score between the two groups is significant at 1%. These results tend to suggest to us that internet users have a lower risk of depression than non-users.

**Table 2:** Summary statistics.

**Variables**	**Mean**	**S.D**.	**Mean of non-users (A)**	**Observations**	**Mean of users (B)**	**Observations**	**Difference (A–B)**
Depression	8.709	6.443	9.061	15,092	6.374	2,273	2.687^***^
Age	61.37	9.589	62.269	15,092	55.366	2,273	6.903^***^
Male	0.475	0.499	0.463	15,092	0.553	2,273	−0.090^***^
Urban	0.204	0.403	0.166	15,092	0.451	2,273	−0.285^***^
Primary	0.653	0.476	0.712	15,092	0.259	2,273	0.454^***^
Junior	0.223	0.416	0.203	15,092	0.357	2,273	−0.155^***^
Senior	0.124	0.330	0.085	15,092	0.384	2,273	−0.299^***^
Family size	3.197	1.851	3.212	15,092	3.092	2,273	0.121^***^
Married	0.797	0.402	0.791	15,092	0.839	2,273	−0.048^***^
Sleep time	6.200	1.971	6.182	15,092	6.317	2,273	−0.136^***^
Vision	0.283	0.451	0.261	15,092	0.432	2,273	−0.171^***^
Agricultural work	0.481	0.500	0.506	15,092	0.313	2,273	0.193^***^
Retirement	0.179	0.383	0.160	15,092	0.309	2,273	−0.150^***^
Subsistence allowance	0.073	0.260	0.081	15,092	0.020	2,273	0.061^***^
Drinking	0.267	0.442	0.253	15,092	0.358	2,273	−0.105^***^
Smoking	0.416	0.493	0.412	15,092	0.439	2,273	−0.027^**^

The standard t-test was used to calculate the average difference between the two groups.

^**^ and ^***^ indicate the significance levels of 5 and 1%, respectively.

There are also differences in covariates between internet users and non-users. Generally, the young, male, highly educated, and married respondents are more likely to use the internet than their counterpartners. Respondents with an urban Hukou have a higher probability of using the internet than those respondents with a rural Hukou. Internet users often have a smaller family size and better eyesight than non-users. Besides, internet users tend to get retired, less likely to have agricultural work, and more likely to receive basic subsistence allowance from the government. Internet users also drink and smoke more often than non-users.

The above results give a preliminary indication about internet use to prove that it is highly associated with the elderly's depression status. However, since there are also significant differences in many covariates, we cannot confidently conclude that there is a causal effect of internet use on the incidence of depression in the elderly. It is therefore necessary to further address the concern of potential selection bias in the rest of the analysis.

### Determinants of internet use

Table 3 reports the determinants of the elderly's choice of internet use from Equation (1). The regression coefficients are reported in column (1). As shown in column (2), we also computed the average marginal effects of the variables. We find that age has a significant and negative impact on the probability of internet use. A 1-year increase in the respondent's age decreases the probability of using the internet by 0.8%. The probability of using the internet for men is 1.5% higher than that for women. The elderly who have an urban Hukou are more likely to use the internet. Compared with the elderly who have an education of primary school or below, those who have a higher level of education are more likely to use the internet, implying that education increases the likelihood of using the internet. Besides, the elderly person who has a larger family is less likely to use the internet, probably because internet use, as a substitution of social communication, is less necessary for the elderly in such large families.

**Table 3:** Determinants of the elderly's choice of internet use.

**Variables**	**(1)**	**(2)**
Age	−0.058^***^	−0.008^***^
	(0.002)	(0.000)
Male	0.110^**^	0.015^**^
	(0.045)	(0.006)
Urban	0.403^***^	0.064^***^
	(0.039)	(0.007)
Junior	−0.968^***^	0.087^***^
	(0.040)	(0.007)
Senior	−0.442^***^	0.209^***^
	(0.039)	(0.012)
Family size	−0.036^***^	−0.005^***^
	(0.008)	(0.001)
Married	−0.048	−0.007
	(0.039)	(0.005)
Sleep time	−0.006	−0.001
	(0.008)	(0.001)
Vision	0.160^***^	0.023^***^
	(0.030)	(0.004)
Agricultural work	−0.241^***^	−0.032^***^
	(0.031)	(0.004)
Retirement	0.270^***^	0.041^***^
	(0.040)	(0.007)
Subsistence allowance	−0.504^***^	−0.049^***^
	(0.081)	(0.005)
Drinking	0.108^***^	0.015^***^
	(0.035)	(0.005)
Smoking	−0.031	−0.004
	(0.043)	(0.006)
Observations	17,365	17,365
Pseudo *R*^2^	0.251	0.251

The regression coefficients are reported in column (1). The average marginal effects are reported in column (2). Robust standard errors are given in parentheses.

^**^ and ^***^ indicate the significance levels of 5% and 1%, respectively.

Vision has a significant and positive impact on internet use. It is intuitive that good vision is a prerequisite for using the internet, especially for the elderly who often suffer from eye diseases in China. The elderly who are engaged in agricultural work are less likely to use the internet. Apart from the reason that access to the internet remains limited in a rural area, another reason could be attributed to the fact that farmers may have limited knowledge of internet and may evince less interest in internet use in China.

The retired respondents have a higher probability of using the internet than the respondents who have not retired from active service, probably because the retired have more time available at their disposal for leisure. The elderly who receive subsistence allowance from the government, arguably the poor people, are unlikely to use the internet. Indeed, internet use could cost a lot of money for the poor in China, which excludes the poor elderly from internet use. The elderly who drink alcohol are more likely to use the internet, probably because wine is a luxury good and drinking alcohol may also represent the income level.

### The effect of internet use on depression from PSM

Table 4 reports the effects of internet use on the elderly's depression status from the propensity score matching. Different matching algorithms generate similar results. Overall, the elderly who use the internet have a lower depression score than those who do not use the internet. The size of the effects ranges from −0.916 to −1.042, which accounts for a reduction effect of ~13% in the depression scores. The statistical significances are all set at the 1% level, regardless of the matching algorithms. These results support our expectation that internet use functions to reduce the incidence rate of depression in the elderly group in China.

**Table 4:** The effect of internet use on depression from propensity score matching (PSM).

**Matching algorithm**	**Treated**	**Control**	**ATT**	**S.E**.	**T-statistics**	**Rosenbaum bounds**
NN(1)	6.412	7.455	−1.042	0.208	−5.02^***^	1.75–1.76
NN(5)	6.412	7.395	−0.983	0.167	−5.88^***^	2.14–2.15
NN(10)	6.412	7.379	−0.967	0.164	−5.88^***^	2.20–2.21
Radius	6.412	7.328	−0.916	0.165	−5.53^***^	2.21–2.22
Kernel	6.374	7.319	−0.946	0.156	−6.08^***^	2.33–2.34

Authors' own calculation.

^***^ indicates the significance level of 1%.

Several tests are conducted to support the validity of our results. First, Supplementary Figure A1 shows that the overlap of propensity scores between internet users and non-users is sufficiently large, implying that most internet users can find a matching partner. Thus, our results can be generalized to the whole sample. Second, results from the balancing test (Supplementary Table A2) show that there is no significant difference in covariates between internet users and non-users after matching. Third, the sensitivity tests of the results report that the Rosenbaum bounds are all larger than 1.75, which implies that the significance of our results is not sensitive to omitted variables.

In this paper, we also test the heterogeneous effects across age. Specifically, we split the sample into two groups, namely the elderly aged 60 or below and the elderly aged over 60. We then estimate the impact of internet use on depression status using the PSM. Table 5 shows that, on average, the impact of internet use on depression status for the elderly aged 60 or below ranges from −0.647 to −0.790, while for the elderly aged over 60 it ranges from −1.264 to −1.987. That is, the reduction effect of internet use on the depression status is larger for the even older groups.

**Table 5:** Heterogeneity in health impacts of internet use by age.

**Matching algorithm**	**Age** ≤ **60**				**Age** > **60**			
	**ATT**	**S.E**.	**T-statistics**	**Rosenbaum bounds**	**ATT**	**S.E**.	**T-statistics**	**Rosenbaum bounds**
NN(1)	−0.671	0.242	−2.77^***^	1.61–1.62	−1.987	0.406	−4.90^***^	3.00–3.01
NN(5)	−0.699	0.200	−3.49^***^	1.98–1.99	−1.390	0.326	−4.26^***^	3.23–3.24
NN(10)	−0.647	0.197	−3.28^***^	1.98–1.99	−1.264	0.320	−3.95^***^	3.19–3.20
Radius	−0.679	0.197	−3.45^***^	2.04–2.05	−1.336	0.328	−4.07^***^	3.33–3.34
Kernel	−0.790	0.183	−4.31^***^	2.55–2.56	−1.535	0.281	−5.46^***^	4.31–4.32

Authors' own computation.

^***^ indicates the significance level of 1%.

To test the heterogeneous effects across gender, we estimate the effects of internet use on the depression status for men and women separately. Table 6 reports the results. We find that the effect of internet use on depression score for the male elderly ranges from −0.684 to −0.851, using different matching algorithms, while the effect of internet use on depression score for the female elderly ranges from −0.977 to −1.028.

**Table 6:** Heterogeneity in health impacts of internet use by gender.

**Matching algorithm**	**Male**				**Female**			
	**ATT**	**S.E**.	**T-statistics**	**Rosenbaum bounds**	**ATT**	**S.E**.	**T-statistics**	**Rosenbaum bounds**
NN(1)	−0.691	0.255	−2.71^***^	1.72–1.73	−1.028	0.328	−3.13^***^	1.89–1.90
NN(5)	−0.684	0.212	−3.23^***^	2.09–2.10	−1.001	0.276	−3.63^***^	2.26–2.27
NN(10)	−0.755	0.210	−3.60^***^	2.21–2.22	−1.044	0.272	−3.84^***^	2.34–2.35
Radius	−0.730	0.211	−3.47^***^	2.22–2.23	−0.977	0.272	−3.60^***^	2.32–2.33
Kernel	−0.851	0.191	−4.46^***^	2.57–2.58	−1.012	0.249	−4.06^***^	2.55–2.56

Authors' own computation.

^***^ indicates the significance level of 1%.

We also test the heterogeneous effects across occupation. We split the sample into two groups according to whether the elderly has any agricultural work or not. Table 7 reports the results. We find that the effects of internet use on depression status for the elderly with agricultural work range from −0.970 to −1.080. For the elderly who do not have any agricultural work, the effects range from −0.742 to −0.888.

**Table 7:** Heterogeneity in health impacts of internet use by work.

**Matching algorithm**	**Agricultural work**				**Non-agricultural work**			
	**ATT**	**S.E**.	**T-statistics**	**Rosenbaum bounds**	**ATT**	**S.E**.	**T-statistics**	**Rosenbaum bounds**
NN(1)	−1.010	0.340	−2.97^***^	2.05–2.06	−0.854	0.253	−3.37^***^	1.74–1.75
NN(5)	−1.062	0.279	−3.80^***^	2.54–2.55	−0.742	0.211	−3.52^***^	2.08–2.09
NN(10)	−1.080	0.273	−3.95^***^	2.58–2.59	−0.752	0.208	−3.62^***^	2.14–2.15
Radius	−1.023	0.269	−3.80^***^	2.61–2.62	−0.768	0.212	−3.62^***^	2.17–2.18
Kernel	−0.970	0.254	−3.82^***^	2.70–2.71	−0.888	0.197	−4.52^***^	2.44–2.45

Author' computation.

^***^ indicates the significance level of 1%.

### The effect of internet use on depression from ESR

Table 8 reports the results from the endogenous switching regression (ESR) model. The *F*-statistic from tests on the strength of the instrumental variable in the selection function is 1,335.285 (*P*-value = 0.000), which exceeds the critical value of 10. This result implies that the concern of a weak instrumental variable should not be a problem in our work. Table 8 shows that the ESR model reports negative but larger effects of internet use on the elderly's depression scores. All the effects are statistically significant at 1% level. These results highlight the importance of addressing selection bias from unobserved confounds. On average, internet use reduces the elderly's depression scores by 3.370 points, which accounts for an approximate reduction of 37.19% from the average depression scores of the non-users.

**Table 8:** The effect of internet use on depression using the endogenous switching regression (ESR) model.

	**ATT**	**S.E**.	**T-statistics**
Main effects	−3.370	0.069	−48.768^***^
Age ≤ 60	−3.251	0.077	−42.090^***^
Age > 60	−3.772	0.148	−25.571^***^
Male	−3.145	0.084	−37.400^***^
Female	−3.648	0.099	−36.732^***^
Agricultural work	−3.627	0.122	−29.625^***^
No agricultural work	−3.252	0.080	−40.480^***^

Author' own computation. The F-statistic of the strength test of instrumental variables in the selection function is 1,335.285 (P-value = 0.000). T-statistics from tests over the differences in the effects of internet use on depression are −11.12 (P-value = 0.000) for age, −12.85 (P-value = 0.000) for gender, and 8.74 (P-value = 0.000) for agricultural work.

^***^ indicates the significance level of 1%.

Table 8 also shows that the general pattern derived from PSM still holds. That is, the effect of internet use on the depression status is larger for the older, female elderly, or the elderly who have agricultural work. Specifically, the effects of internet use on the depression status for the older and younger elderly are −3.772 and −3.251, respectively. The effects of internet use on the depression status for the male and female elderly are −3.145 and −3.648, respectively. While the effect on the elderly who have agricultural work is −3.627, for the elderly who have no agricultural work, it is −3.252. The *T-*statistics from tests over differences in the effects between different ages, genders, and employment status are −11.12, −12.85, and 8.74, respectively. The corresponding *P*-values are all smaller than 1%, which supports the heterogeneous effects of internet use.

## Discussion

This paper estimates the impact of internet use on the elderly's depression scores, using the data from the China Health and Retirement Longitudinal Survey in 2018. To address the concern of potential selection bias, the propensity score matching (PSM) and the endogenous switching regression (ESR) model are employed. Our main finding is that internet use significantly reduces the elderly's depression scores by 3.370 points, which accounts for a reduction of ~37.19% of the average depression scores of the elderly who do not use the internet.

The health effect of internet use has been a big concern for scientists and policymakers alike. Generally, there are three different opinions that are voiced about the health effect of internet use, i.e., negative, neutral, and positive (52). For example, some studies voice skepticism on the internet use, based on its negative health effects on the teenagers (25, 26). Some other studies find that the internet use is irrelevant to the health and living habits of the elderly (48). More studies, however, find that internet use is positively associated with the mental health of the elderly (47, 53). Our work supports the literature on the positive effect of internet use by linking internet use to the reduction in the elderly's depression scores, and by focusing on causality rather than association.

Our work is related to the literature on the effect of internet use and the determinants of elderly's mental health. Previous studies have investigated how the use of internet may affect the adoption of an environmentally friendly behavior (54, 55), household welfare and wellbeing (56), and agricultural production and marketing performance (57, 58). Some other studies have made effort to understand the diversity of elderly's mental health (11, 42, 59, 60). Yet, there is still limited knowledge about the relationship between the behavior of internet use and mental health.

As exceptions, two existing studies find a strong correlation between internet use and life satisfaction (61, 62). Another study finds that frequent internet use makes people feel lonely (63). These studies, however, only focus on some aspects of depression and investigate correlation rather than causality. Different from these studies, a recent work estimated the causal effect of internet use on residents' depression status (47), even though causality cannot be fully identified due to a flaw in selected method. Using an instrumental variable-based approach, another work finds a negative effect of internet access on young women's mental health (64). Our work complements the above strand of literature by measuring depression with a more comprehensive indicator. We also provide another effort to identify the causal effect of internet use on the elderly. The positive effects of internet use on the elderly's mental health in our research contradict with the negative effects of internet use on the young in previous research.

Another benefit of our work to the literature is that we investigated the heterogeneous effects of internet use. Existing studies on the health effects of internet use on the elderly's depression status often estimate its average effects (47, 53). But our work has brought out the fact that the health effects of internet use are larger for the older, female elderly, or those who have agricultural work than those for their counterpartners. These results provide supportive evidence for the opinion that internet use can function as a cost-effective type of social participation (65). Indeed, internet use, as the substitution of social participation, should be more important for even the older elderly, who have more difficulty in engaging in social participation (66). In addition, in the Chinese society, women often have less time for social participation than men (67). As a quick and low-cost approach to connect and communicate with other people, internet should have a larger substitution effect for women than for men. As regards occupation, working in the agricultural sector often implies that the elderly are probably living in a rural area, where the access to social participation is limited (68). On the contrary, the elderly who do not live in a rural area may have more choices for engaging in social participation other than surfing the internet.

Despite the larger health effect of internet use on the older, female, or the elderly who have agricultural work, the probability of internet use by these elderly is significantly lesser. Such results seem to reflect an awkward situation, that is, the potentially best beneficiaries have no access to the internet in China, which may have important implications for policymakers. There are also other determinants of internet use, such as education, family size, eye health, living habits, and so on, which are mostly in line with those of the existing studies (69, 70).

## Conclusion

In aging countries, i.e., in countries of the world whose population is slowly aging, the prevalence of mental health problems in the elderly is challenging the sustainability of the societies at large. In this paper, we investigate the role of internet use in reducing the elderly's depression. We find that the internet use significantly reduces the incidence of depression in the elderly, especially for those who are older, female, and have agricultural work. Besides, the choice of internet use by the elderly is determined by age, gender, education, family size, eye health, working status, living habits, and some other individual or family characteristics.

Our findings have important policy implications. To improve the mental health of the elderly, the government should put in more effort to the adoption of the internet by the elderly. In particular, the needs of the elderly who are older, female, or have agricultural work should be satisfied. Specifically, restrictions imposed on the elderly to access the internet should be removed. More subsidies should be given to the poor elderly groups. Considering that a great number of the elderly have poor eyesight, it is necessary to create elderly oriented electronic devices and gadgets to motivate the elderly to continue to use the internet.

Our work may bring to light a few limitations. Due to data limitation, internet use is measured as a dummy that merely reflects whether the elderly use the internet or not. However, the frequency and the duration of internet use, which may have different impacts on the elderly's health, are not included and so ignored in our analysis. In addition, the purpose of internet use, e.g., to seek information, to communicate with friends, or to entertain themselves, may also lead to different impacts on the elderly. Thus, to gain a better understanding of the benefits of internet use, future work should investigate further the health effects of the frequency, the duration, and the purpose of internet use on the elderly.

## Data availability statement

The original contributions presented in the study are included in the article/Supplementary material, further inquiries can be directed to the corresponding author.

## Author contributions

HG: conceptualization, formal analysis, and writing-original draft. SF: methodology, writing-review and editing, and resources. ZL: conceptualization, methodology, validation, writing-review and editing, and supervision. All authors contributed to the article and approved the submitted version.
