# Supplementary material for: The temperature of internet: Internet use and depression of the elderly in China
Source: Front Public Health. 2022 Dec 21;10:1076007. doi: 10.3389/fpubh.2022.1076007 (PMC9811204; doi:10.3389/fpubh.2022.1076007)
Supplement: Supplementary file 1 [file Data_Sheet_1.docx]

**Appendix**

Table A1 CES-D Depression questionnaire

| Question code | Questions |
| --- | --- |
| DC009 | In the past week, I was bothered by things that don’t usually bother me. |
| DC010 | In the past week, I had trouble keeping my mind on what I was doing. |
| DC011 | In the past week, I felt depressed. |
| DC012 | In the past week, I felt everything I did was an effort. |
| DC013 | In the past week, I felt hopeful about the future. |
| DC014 | In the past week, I felt fearful. |
| DC015 | In the past week, I could not sleep well. |
| DC016 | In the past week, I was happy. |
| DC017 | In the past week, I felt lonely. |
| DC018 | In the past week, I could not get “going”. |
| Note: This is from the original questionnaire for the CHARLS survey. For each question, the answer options are (1) rarely or none of the time, (2) some or a little of the time, (3) occasionally or a moderate amount of the time, and (4) most or all of the time. | |

Table A 2 Results of the balancing tests

| Variable | Before matching | | |  | After matching | | |
| --- | --- | --- | --- | --- | --- | --- | --- |
|  | Treated | Control | T-statistics |  | Treated | Control | T-statistics |
| Age | 55.366 | 62.269 | -32.98*** |  | 55.554 | 55.431 | 0.57 |
| Male | 0.553 | 0.463 | 8.03*** |  | 0.554 | 0.552 | 0.16 |
| Urban | 0.451 | 0.166 | 32.35*** |  | 0.434 | 0.434 | 0.01 |
| Junior | 0.357 | 0.203 | 16.64*** |  | 0.368 | 0.378 | -0.72 |
| Senior | 0.384 | 0.085 | 42.30*** |  | 0.365 | 0.352 | 0.91 |
| Family size | 3.092 | 3.213 | -2.91*** |  | 3.105 | 3.096 | 0.19 |
| Married | 0.839 | 0.791 | 5.29*** |  | 0.841 | 0.841 | -0.04 |
| Sleep time | 6.318 | 6.182 | 3.06*** |  | 6.316 | 6.330 | -0.29 |
| Vision | 0.432 | 0.261 | 17.01*** |  | 0.421 | 0.415 | 0.43 |
| Agricultural work | 0.313 | 0.506 | -17.30*** |  | 0.322 | 0.328 | -0.42 |
| Retirement | 0.309 | 0.160 | 17.51*** |  | 0.304 | 0.316 | -0.92 |
| Subsistence allowance | 0.020 | 0.081 | -10.46*** |  | 0.020 | 0.020 | 0.18 |
| Drinking | 0.358 | 0.253 | 10.56*** |  | 0.353 | 0.342 | 0.79 |
| Smoking | 0.439 | 0.412 | 2.43** |  | 0.441 | 0.441 | -0.01 |
| Notes: Authors’ own calculation. The results are obtained from the nearest neighbor matching with ten partners. Matching quality with other algorithm produces close results. ** indicates a significance level of 5%, *** indicates a significance level of 1%. | | | | | | | |


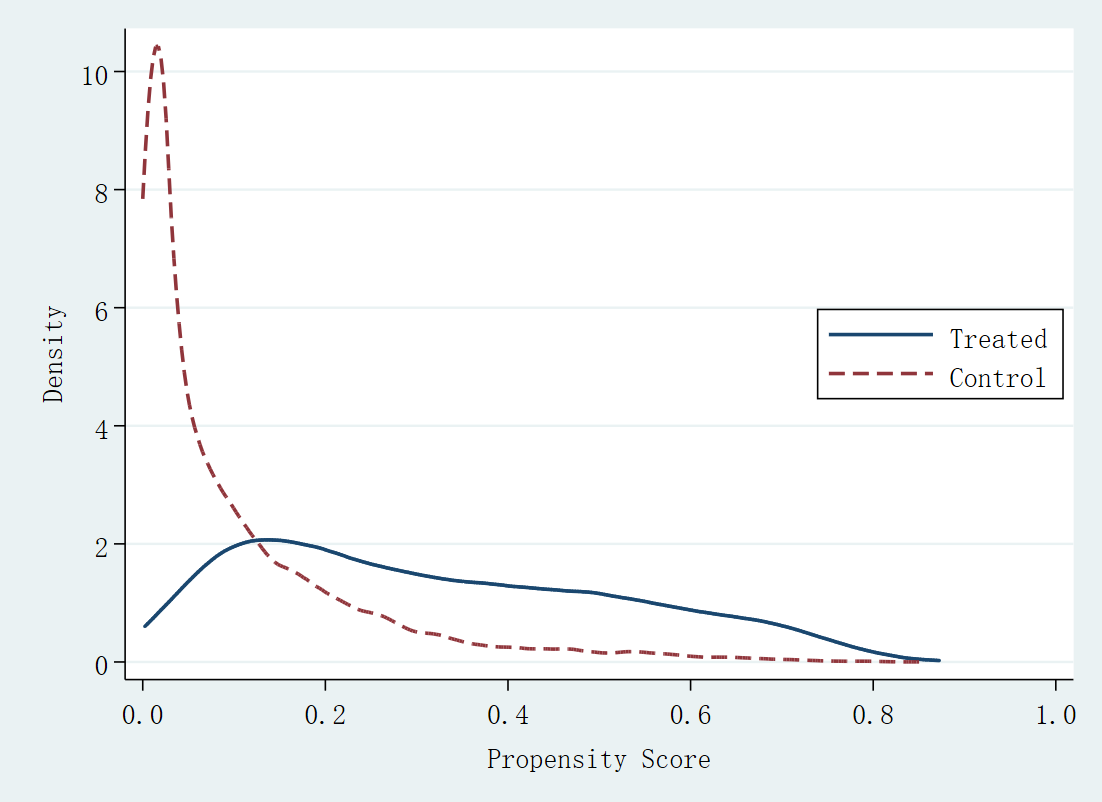


Figure A1 The distribution of estimated propensity scores.
